# A unified model for regulating lipoprotein lipase activity

**DOI:** 10.1016/j.tem.2024.02.016

**Published:** 2024-03-23

**Authors:** Ren Zhang, Kezhong Zhang

**Affiliations:** 1 Center for Molecular Medicine and Genetics, Wayne State University School of Medicine, Detroit, MI 48201, USA

## Abstract

The regulation of triglyceride (TG) tissue distribution, storage, and utilization, a fundamental process of energy homeostasis, critically depends on lipoprotein lipase (LPL). We review the intricate mechanisms by which LPL activity is regulated by angiopoietin-like proteins (ANGPTL3, 4, 8), apolipoproteins (APOA5, APOC3, APOC2), and the cAMP-responsive element-binding protein H (CREBH). ANGPTL8 functions as a molecular switch, through complex formation, activating ANGPTL3 while deactivating ANGPTL4 in their LPL inhibition. The ANGPTL3-4-8 model integrates the roles of the aforementioned proteins in TG partitioning between white adipose tissue (WAT) and oxidative tissues (heart and skeletal muscles) during the feed/fast cycle. This model offers a unified perspective on LPL regulation, providing insights into TG metabolism, metabolic diseases, and therapeutics.

## Triglyceride partitioning, a fundamental process that helped human ancestors to survive

TGs have been pivotal in human evolution owing to their dense and efficient energy content. Composed of glycerol and three fatty acids, these molecules offer over twice the amount of energy per gram compared to carbohydrates or proteins. For early humans, who were subject to cycles of abundance and scarcity, TGs stored in adipose tissue were a crucial energy reserve during lean times, ensuring survival [1]. When the body enters a fasting state – between meals or during extended periods without food – stored TGs are utilized to provide sustained energy for muscles. This mechanism supports physical activity when glucose levels are low, and sustains essential bodily functions and overall metabolism.

In the bloodstream, TGs are transported by lipoproteins, notably chylomicrons (CMs) and very low density lipoproteins (VLDLs). Following food intake, CMs form in the small intestine following the absorption of dietary fats, primarily consisting of TGs. CMs represent the largest and least dense of the lipoproteins. VLDLs, which are produced in the liver primarily during fasting, carry TGs from the liver to other tissues [2]. Both CMs and VLDLs play vital roles in lipid metabolism by distributing fats to where they are needed in the body.

TG partitioning refers to the distribution or allocation of TGs within the body, and specifically it involves the process of transferring circulating TGs into WAT after food intake and transferring them to oxidative tissues (heart, skeletal muscle) during fasting. Proper regulation of TG storage and utilization is essential for maintaining energy balance and overall health. When the TG partitioning process malfunctions, it can lead to metabolic disorders. Hypertriglyceridemia, obesity, and insulin resistance correspond to an excess of TGs in the bloodstream, adipose tissue, and muscles, respectively. Understanding TG partitioning is crucial for managing metabolic health and diseases, including hyperlipidemia, obesity, and cardiovascular diseases. TG partitioning is critically dependent on the enzyme LPL. Significant progress has been made in deciphering the mechanisms of LPL activity regulation, which is orchestrated by a diverse set of regulators in a tissue-specific manner. The current review integrates these regulators into the ANGPTL3-4-8 model, which, by offering a unified perspective, illuminates the interconnected and interdependent relationships among LPL regulators throughout the feed/fast cycle.

## LPL, a gatekeeper of tissue-specific TG delivery

The discovery of LPL dates to 1943, with Paul Hanh’s intriguing observation in dogs that a mysterious ‘clearing factor’ dissolved fat droplets (CMs) after meals [3]. A crucial leap forward came in 1955 when Edward Korn purified this factor, identified it as a dedicated enzyme, and named it lipoprotein lipase [4]. LPL is a pivotal enzyme in the body’s fat metabolism and is found predominantly in adipose tissue and muscle cells. Its primary function involves breaking down TG – carried by circulating lipoproteins such as CMs and VLDLs – into glycerol and fatty acids, with the latter being either stored in WAT or utilized by oxidative tissues for energy.

The activity of LPL significantly influences the movement and distribution of fats within the body, known as TG partitioning flux. Higher LPL activity in specific tissues, such as WAT, promotes increased uptake of fatty acids from circulating lipoproteins and directs them for storage. Conversely, reduced LPL activity may lower fat storage and increase fat utilization for energy in tissues such as muscle [5–12]. LPL deficiency, a rare genetic disorder, results in decreased or absent LPL enzyme activity. This deficiency leads to elevated blood TG levels, causing complications such as pancreatitis and potentially eruptive xanthomas [13].

Essentially, LPL acts as a gatekeeper of tissue-specific fat delivery, through either utilization or storage, to dynamically balance TG distribution throughout the body. Its role varies depending on the feeding/fasting state of the body. Postprandial LPL activity increases in WAT but declines in oxidative tissues, enabling the uptake of TG into WAT. Conversely, fasting LPL activity declines in WAT, but rises in oxidative tissues, ensuring that TGs are taken up by the latter to meet the energy needs of the body [8,14]. LPL activity is profoundly regulated by apolipoproteins and ANGPTLs [6,10,12,14,15].

## Regulators of LPL activity

Two major categories of LPL activity regulators are apolipoproteins and ANGPTLs, where the former includes APOA5, APOC3, and APOC2, and the latter includes ANGPTL3, 4, and 8 (A3, 4, 8). Other regulators include CREBH and GPIHBP1.

### Apolipoprotein A5 (APOA5)

APOA5 plays a crucial role in lipid metabolism and the regulation of plasma TG levels [16]. Synthesized mainly in the liver, APOA5 interacts with lipoproteins such as CMs and VLDLs and influences their metabolism. Its primary function involves the regulation of TG levels by positively regulating LPL activity, enhancing the breakdown of TG-rich particles, and promoting their clearance from the bloodstream. APOA5 deficiency is associated with elevated plasma TG levels, which can increase the risk of cardiovascular diseases [16]. Therefore, APOA5 is considered to be a significant factor in lipid metabolism and in maintaining healthy TG levels in the body.

### APOC3

APOC3 is found on the surface of various lipoproteins, particularly VLDLs [17]. Its primary role involves regulating TG levels in the bloodstream by influencing lipid metabolism. By negatively regulating LPL activity, APOC3 reduces the clearance of TG-rich particles such as VLDLs, leading to elevated TG levels in the blood. High levels of APOC3 are associated with increased cardiovascular risk owing to its role in promoting the accumulation of TG in the circulation. Therefore, APOC3 is considered to be a significant factor in lipid metabolism and is linked to conditions including hypertriglyceridemia and cardiovascular diseases [17].

### APOC2

APOC2 is primarily associated with CMs and VLDLs. By activating LPL, APOC2 facilitates the conversion of TGs into fatty acids [17,18]. APOC2 deficiency is a rare genetic disorder characterized by insufficient or absent levels of APOC2 protein [17,18]. This deficiency leads to impaired activation of LPL, and individuals with APOC2 deficiency experience severe hypertriglyceridemia [17,18].

### ANGPTL3 (A3)

A3 is primarily produced in the liver and plays a significant role in controlling TG and cholesterol levels in the bloodstream [19–21]. A3 functions by inhibiting LPL and endothelial lipase (EL), which are involved in the breakdown of TG and high-density lipoproteins (HDL), respectively, in peripheral tissues. By inhibiting these enzymes, A3 reduces the breakdown of TGs from circulating lipoproteins, such as CMs and VLDLs, and increases the levels of these lipoproteins in the blood. Research and clinical studies on A3 suggest that inhibiting its activity offers a potential therapeutic approach for managing dyslipidemia, a condition characterized by abnormal lipid levels in the blood, and for reducing the risk of cardiovascular diseases [22–24]. Therapies targeting A3 are being explored as a means to regulate lipid metabolism and potentially decrease the risk of atherosclerosis and related cardiovascular complications.

### ANGPTL4 (A4)

A4 was identified as a novel ANGPTL family member induced by fasting via the peroxisome proliferator-activated receptor (PPAR) in adipocytes [25–27]. A4 acts as a pivotal regulator of fat metabolism by controlling the action of LPL. A4, as a potent LPL inhibitor [21,28], plays an important role in regulating LPL activity under fasting conditions [29]. Consistently, A4 knockout (KO) mice and wild-type (WT) mice treated with a neutralizing antibody have decreased plasma TGs and increased post-heparin plasma LPL activity [21,28,30–32]. A4 plays a crucial role in LPL-mediated TG uptake by WAT because both whole-body A4 KO and adipose-specific KO mice show reduced TG uptake into WAT [33,34]. Of note, although A4 is abundant in both WAT and the liver, A4 in WAT, but not in the liver, majorly regulates TG partitioning [34,35]. Multiple mechanisms have been proposed for how A4 inhibits LPL [36–38]. For example, A4 promotes the unfolding of LPL, leading to the cleavage and subsequent degradation of LPL [12,36,37]. A4 can also reduce the affinity of LPL for binding to GPIHBP1 [39]. A4 mediates fat storage driven by the gut microbiota [40]. Furthermore, genetic variations in A4 are consistently associated with lipid profiles in genome-wide association studies (GWAS) [41–43]. Briefly, WAT A4 primarily functions as an autocrine regulator of LPL during fasting by inhibiting LPL activity and promoting its degradation, and targeting A4 emerges as a promising therapeutic approach for managing dyslipidemia and cardiovascular diseases.

### ANGPTL8 (A8)

The functional roles in lipid metabolism of a previously uncharacterized gene, *Gm6484*, were reported by multiple groups in 2012 under various names, such as lipasin, which was later officially known as ANGPTL8 [44–46]. A8 expression is specific to the liver and fat and is highly induced by food intake [44–48]. A8 is also abundant in brown fat, where its expression in induced by cold exposure [49]. Recent data convincingly showed that circulating A8 is mainly from secretion by the liver, and not from WAT [50]. A8 can form a complex with A3, activating its LPL inhibitory activity [51–53]. Liver A8 forms the A3/8 complex, which, after being secreted into the circulation, inhibits oxidative-tissue LPL in an endocrine manner [8,14]. A8 can form a complex with A4, canceling A4’s inhibition of LPL [52,54]. Mice with A8 hepatic overexpression exhibit increased TG levels, and A8 KO mice exhibit lower TG levels [44,45,55,56]. CRISPR/Cas9-mediated A8 KO in rats suppresses plasma TGs and adiposity [57]. Consistently, injection of an A8-specific monoclonal antibody in mice reduces plasma TGs by a mechanism involving increased postprandial activity of cardiac LPL [55]. A8 blockade with a monoclonal antibody (REGN3776, generated by Regeneron Pharmaceuticals) promotes TG clearance and weight loss in mice, and administration of this antibody normalizes plasma TG levels in dyslipidemic cynomolgus monkeys [58]. Furthermore, an antibody specific for the A3/8 complex effectively blocks A3/8 inhibition of LPL [59,60] and dramatically lowers TG levels in mice [60] and humans [61].

In humans, A8 sequence variations have been demonstrated to be associated with lipid profiles by GWAS. The SNP rs2278426, representing the R59W variant, has been associated with lipid traits in multiple independent populations [46,62]. The SNP rs145464906 leads to a truncated A8 [120 amino acids (aa), the full-length protein being 198 aa]. Carriers of the T allele had lower TG and higher HDL-C levels than non-carriers [63], and this result was confirmed by analysis based on an independent cohort in the UK Biobank [64]. The SNP rs760351239 leads to a truncated A8 (130 aa), and carriers of the T allele had 24.0 mg/dl lower TG and 9.1 mg/dl higher HDL-C levels [64].

Physiologically, the circulating levels of A8 in humans were found to be decreased by overnight fasting [50] and increased 2 h following a defined meal [65]. Food intake increases circulating levels of A8, A3/8, and A4/8, and these levels are decreased by fasting [52,65] or by exercise [66]. In summary, overwhelming evidence from both loss- and gain-of-function studies in mice as well as human GWAS has demonstrated that A8 is a feeding-induced hepatokine that is a potent regulator of lipid metabolism by regulating LPL activity through forming complexes with A3 and A4. The association of A8 levels with various pathological conditions and its non-metabolic roles, such as in inflammation, are discussed elsewhere [67–70].

### CREBH

CREBH (cAMP responsive element-binding protein, hepatocyte-specific), a transcription factor primarily found in the liver, regulates lipid metabolism, particularly during fasting or metabolic stress [71]. It plays a key role in the regulation of lipid metabolism by influencing the expression of genes involved in these processes [72,73]. CREBH activation is often associated with upregulation of genes related to lipid metabolism, such as those involved in TG synthesis and transport [74]. During fasting or conditions of metabolic stress, CREBH is activated to promote TG lipolysis and fatty acid oxidation. This process is part of the body’s response to provide energy sources, particularly fatty acids, to peripheral tissues such as muscles. It serves as a crucial transcription factor in coordinating the response of the liver to metabolic demands and stressors. Patients with CREBH deficiency exhibit hypertriglyceridemia as a result of reduced LPL activity [75–77]. We recently found that CREBH can be cleaved to release its C-terminal fragment (CREBH-C), which is secreted into the circulation where it acts as a hepatokine to regulate LPL activity [78].

### GPIHBP1

GPIHBP1 (glycosylphosphatidylinositol-anchored high-density lipoprotein-binding protein 1) is crucial for transporting LPL to the endothelial surface. It anchors LPL to the endothelial cells lining blood vessels, thereby facilitating LPL in breaking down TG in circulating lipoproteins [79–81]. This process is essential for providing fatty acids to tissues for energy or storage and for maintaining the lipid balance of the body. Of note, the LPL that GPIHBP1 transports into capillaries can detach and move into the endothelial cell glycocalyx, where it is also functional [82]. Indeed, recent data suggest that LPL can adopt different quaternary structures when not bound by GPIHBP1 in the capillary [83]. GPIHBP1 deficiency is a rare genetic condition that disrupts the proper anchoring of LPL to endothelial cells in blood vessels [84–87]. As a result, individuals with GPIHBP1 deficiency experience elevated TG levels, similar to those seen in LPL deficiency. GPIHBP1 can function as a platform where LPL interacts with its regulators including apolipoproteins and ANGPTLs [88–90].

## Emerging discoveries in LPL regulation

LPL, discovered eight decades ago, has been the subject of extensive research to unveil its intricate biology and regulatory mechanisms. The process of TG partitioning is clear: postprandial LPL activity increases in WAT but decreases in oxidative tissues, thereby directing TG for storage in WAT. Conversely, fasting LPL activity diminishes in WAT but rises in oxidative tissues, channeling TG to the latter [91]. Understanding the mechanisms that determine the tissue-specific activity of LPL during the feed/fast cycle carries significant implications for human health and metabolic diseases.

Research in the past decades has clearly shown that two categories of proteins profoundly affect LPL activity – apolipoprotein and ANGPTLs. As for apolipoproteins, APOC3 negatively whereas APOA5 positively regulates LPL activity, while APOC2 is an LPL activator. A3 and A4 negatively regulate LPL activity but display distinct tissue specificity and nutritional regulation. A8 functions as a molecular switch activating liver A3 while deactivating WAT A4 in their LPL inhibition. Additionally, CREBH, a fasting-induced, liver-enriched transcription factor, positively regulates LPL activity. All these results are supported by vast amounts of solid data in both humans and animal models with loss- and gain-of-function studies. However, the exact relationships among LPL, WAT, oxidative tissues, APOA5, APOC3, APOC2, A3, A4, A8, and CREBH, and their roles during the feed/fast cycle, represent a complex jigsaw puzzle that needs to be solved. We proposed the ANGPTL3-4-8 model [14,55], which provides a framework linking A3, A4, A8, and TG partitioning during the feed/fast cycle. However, three key questions remained unsolved until recently.

(i) The elephant in the room is that both ANGPTLs and apolipoproteins are critical LPL regulators that profoundly affect LPL activity; how, then, are the two classes of proteins coordinated? What are their relations when regulating LPL? Specifically, do they compete or cooperate, and do they interact to regulate LPL?

(ii) During fasting, the circulating A3/8 complex, although reduced in level (~15 ng/ml), is still abundant [52]. What mechanism, then, does the body use to block their activity and allow the restoration of oxidative-tissue LPL activity?

(iii) CREBH is a transcription factor that is enriched in the liver. How does it affect LPL activity in peripheral tissues?

Very recent discoveries [50,92–96], including ours [78], have enabled, for the first time, a unified view of LPL regulation to answer the aforementioned questions.

## An updated ANGPTL3-4-8 model (fasting state)

In healthy subjects, the fasting level of plasma A3/8 is ~15 ng/ml, whereas 2 h following a meal the level rises to ~28 ng/ml [52]. Therefore, fasting reduces circulating A3/8 which, however, still retains a significant presence. This raises the question – how does the body dampen plasma A3/8 and restore oxidative-tissue LPL activity during fasting?

An initial clue lies in APOA5, which was found to be an endogenous A3/8 inhibitor [94]. APOA5 associates with the A3/8 complex and blocks its inhibition of LPL [94]. Later *in vivo* studies demonstrated that the hypertriglyceridemia in APOA5 KO mice results from decreased intracapillary LPL amounts in oxidative tissues and that an anti-A3/8 antibody normalizes circulating TG concentrations in these mice [96]. Of note, fasting upregulates APOA5 expression in the liver, leading to its increased circulating levels.

Patients with CREBH deficiency exhibit elevated circulating TG levels [75–77]. Despite decreased LPL activity in CREBH knockout mice, our understanding of the mechanism behind CREBH activation of LPL remains incomplete. After regulated intramembrane proteolysis, CREBH releases its N-terminus that functions as a transcription factor to activate expression of APOC2, the cofactor for LPL [71–73,77]. We recently discovered that the C-terminal fragment of CREBH (CREBH-C), which is derived from membrane-bound full-length CREBH, is secreted as a hepatokine during fasting [78]. Secreted CREBH-C binds to the A3/8 complex and blocks its ability to inhibit LPL. Forced expression of CREBH-C leads to elevated LPL activity in plasma and metabolic tissues in mice. This study identifies a fasting-induced, secreted protein fragment of CREBH as a hepatokine that stimulates LPL activity and maintains TG balance. Thus, CREBH-C acts as an endogenous liver-derived fasting-induced A3/8 inhibitor.

**Figure 1.**
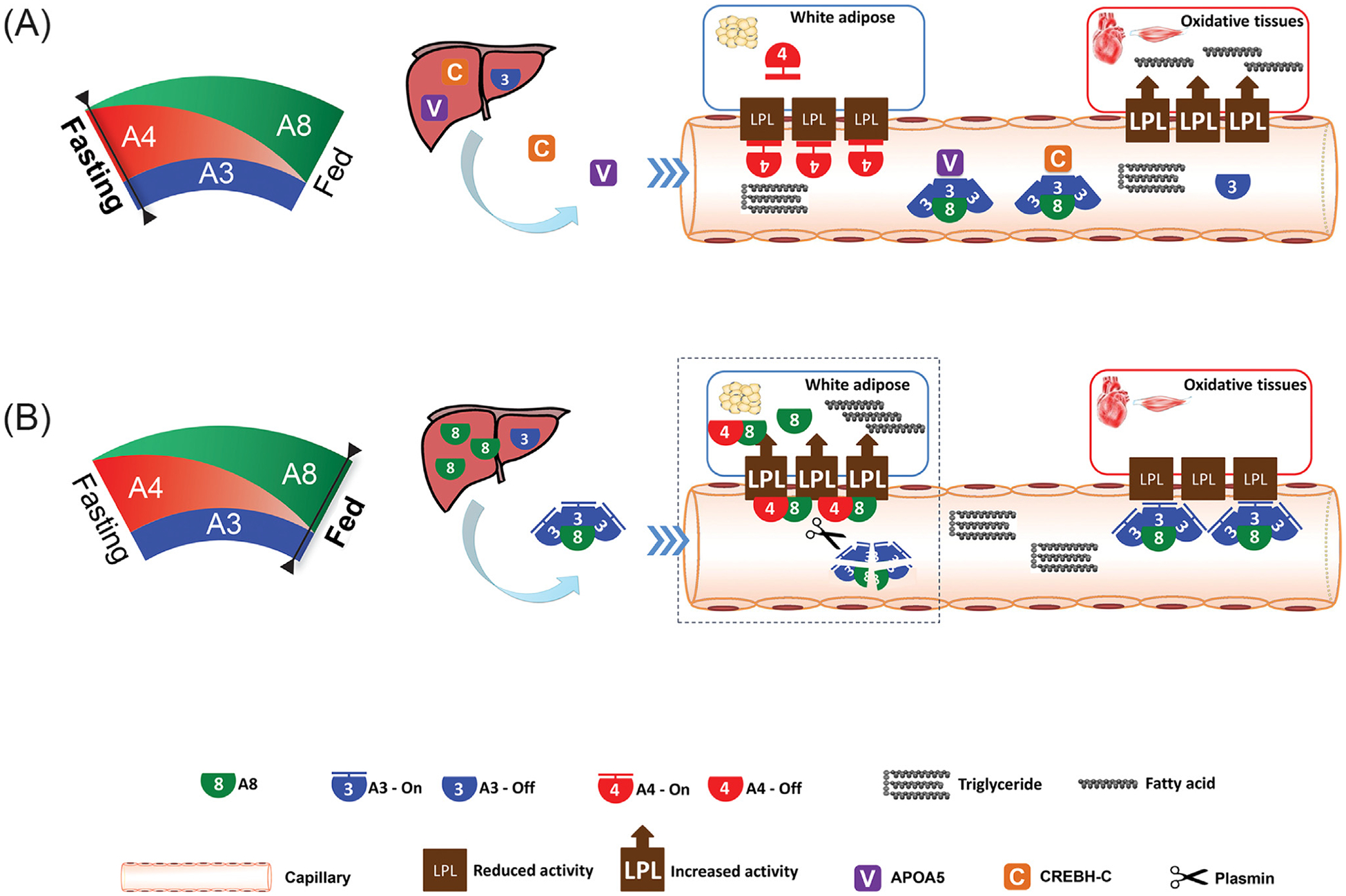
The ANGPTL3-4-8 model. (A) Fasting reduces ANGPTL8 (A8) expression in both liver and white adipose tissue (WAT) but induces WAT A4, which inhibits LPL locally. Fasting triggers the expression and secretion of APOA5 and CREBH-C, which dampen plasma A3/8, thus fully reviving oxidative-tissue LPL. Therefore, fasting LPL activity declines in WAT but increases in oxidative tissues, channeling triglycerides (TGs) to the latter. (B) Conversely, feeding increases liver A8, forming the A3/8 complex (3:1 ratio), which, after being secreted into the circulation, inhibits oxidative-tissue LPL in an endocrine manner. Feeding also increases WAT A8, which forms the A4/8 complex (1:1 ratio) and blocks the inhibitory effect of A4 on LPL. The A4/8 complex, after a series of steps (squared region; detailed in Figure 2), generates plasmin that protects LPL from being inhibited by circulating A3/8. Consequently, postprandial LPL activity surges in WAT but diminishes in oxidative tissues, thereby channeling TGs to the former.

During fasting, the liver and WAT exhibit a rapid reduction in A8 expression, paralleled by decreased A3/8 plasma levels, hence releasing its inhibition of oxidative-tissue LPL. Fasting triggers the expression and secretion of APOA5 and CREBH-C, two endogenous A3/8 inhibitors, blocking circulating A3/8, thus further promoting LPL activity in oxidative tissues. Reduced levels of circulating A3/8 and increased levels of APOA5 and CREBH-C ensure the inhibition of A3/8, thereby allowing the full activity of oxidative-tissue LPL. In WAT, A4 expression drastically increases, locally inhibiting WAT LPL. Consequently, fasting LPL activity declines in WAT but surges in oxidative tissues, channeling TG to the latter (Figure 1).

## An updated ANGPTL3-4-8 model (fed state)

We showed in 2015 that an antibody against A8 lowered plasma TG in mice [55]. Importantly, mice with antibody injection had increased LPL activity in the heart, but not in WAT, suggesting that A8 selectively inhibits LPL activity in the heart [55]. This is a critical piece of information because it suggests that A8 negatively regulates LPL activity in oxidative tissues. A recent study using an antibody against the A3/8 complex demonstrated that, following feeding, the A3/8 complex is located in the endothelium of the mouse heart [95]. These results further strengthen the conclusion that the A3/8 complex inhibits oxidative-tissue LPL.

Hepatic A8 overexpression results in hypertriglyceridemia in mice [44], and this phenotype is dependent on A3 because A8 fails to increase plasma TG in A3 KO mice [46]. It has now become clear that A3 by itself has limited LPL-inhibiting activity, but A8, by forming a complex with A3 (protein ratio 3:1), dramatically increases A3 activity in LPL inhibition [51–53].

Feeding also dramatically induces the level of A8 in WAT, where it forms a complex with A4 (protein ratio 1:1) [52]. A4 by itself is a strong inhibitor of LPL activity, but by forming the A4/8 complex, A8 dampens the inhibitory effect of A4 on LPL [52,54]. In addition, WAT A8 also reduces A4 secretion from adipocytes [50]. Therefore, by blocking A4’s action on LPL and blocking A4 adipocyte secretion, A8 in WAT increases LPL activity. In other words, A8 functions as a molecular switch that turns on A3, but turns off A4, in their LPL inhibition, thereby balancing TG flow in different nutritional states.

**Figure 2.**
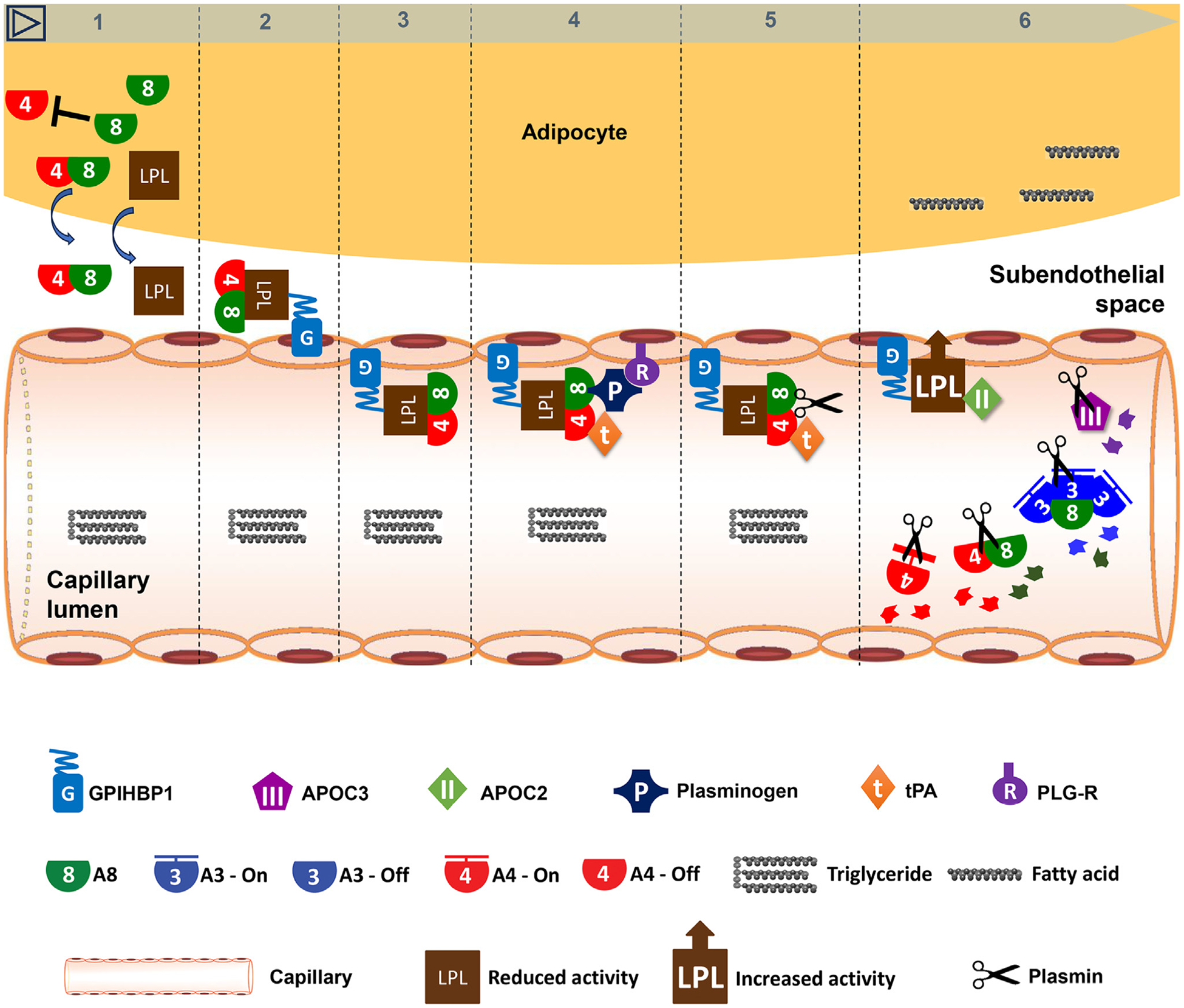
Mechanisms of how ANGPTL4/8 in white adipose tissue (WAT) generates plasmin that cleaves LPL inhibitors in the fed state. Following food intake (1) WAT ANGPTL8 (A8) is dramatically increased. A8 blocks the LPL-inhibiting activity of A4, and A8 also inhibits A4 secretion. (2) The A4/8 complex binds tightly to LPL that is anchored by GPIHBP1. (3) This in turn translocates LPL/A4/8 to the capillary lumen. (4) A4/8 recruits tPA and plasminogen that is bound by its receptor PLG-R. (5) This leads to the generation of plasmin. (6) Plasmin then cuts A4/8, A3/8, A4, APOC3, thus protecting LPL and restoring its activity. Meanwhile, APOC2 functionality is preserved. This intricate choreography ensures active postprandial LPL in WAT.

Following food intake, A8 expression is increased in the liver, forming the A3/8 complex, which, in turn, is secreted into the circulation. In an endocrine manner, A3/8 inhibits LPL specifically in oxidative tissues, making TGs available for uptake. Meanwhile, expression of A8 is dramatically increased in WAT, whereas A4 expression is suppressed. A8 blocks the ability of A4 to inhibit LPL [52,54], and A8 also degrades A4 and suppresses its secretion [50]. A4/8 binds tightly to LPL and is translocated to the capillary lumen. A4/8 recruits plasminogen and tPA, which converts plasminogen into plasmin. Plasmin, in turn, cleaves LPL inhibitors, including A3/8, A4, and APOC3, without affecting the LPL activator APOC2 [92,93]. This series of reactions fully restore WAT LPL activity and enable A3/8 to specifically inhibit oxidative-tissue LPL (Figure 2).

In other words, the A4/8 complex safeguards LPL by guiding it from subendothelial spaces to the luminal surfaces of capillaries in WAT. The A4/8–LPL complex, in turn, interacts with endothelial secreted tPA and luminal plasminogen receptors, enabling the conversion of plasminogen to plasmin. Plasmin cleaves ANGPTL4/8 from LPL and reinstates its activity, and cuts A3/8 near the LPL site. This intricate choreography ensures efficient fat storage after meals (Figure 2).

The discoveries of APOA5 being an A3/8 inhibitor [94] and of A4/8 complex’s biology [54,92,93] are pivotal, as they connect apolipoproteins and ANGPTLs in LPL regulation, reveal how A4/8 rejuvenates postprandial LPL activity in WAT with striking molecular details, and provide mechanistic insights into the tissue-specific inhibition of oxidative-tissue LPL by A3/8. Notably, the data on the A4/8 complex are mainly from *in vitro* experiments, and therefore the results will be strengthened by future *in vivo* studies.

## Explanation of the phenotypes of *Angptl3* KO mice using the ANGPTL3-4-8 model

The phenotypes of A3 KO mice present intriguing insights regarding A3 and LPL biology. A3 KO mice have a more pronounced reduction of plasma TGs during the fed state, despite stable A3 levels [21]. This is easy to explain following the discovery of A8 because A3 is activated by A8, which is feeding-induced. Therefore, in the fed state, the differences between WT and KO mice in plasma A8 levels are more dramatic than in the fasting state, thus explaining the more pronounced TG phenotypes.

However, A3 KO mice also have phenotypes that still have not been satisfactorily explained. First, A3 KO mice display increased LPL activity in WAT [97]. It is expected that A3 KO mice have higher LPL activity in oxidative tissues; it is unexpected, however, that LPL activity is also increased in WAT. Second, although A8 overexpression dramatically increases plasma TGs in WT mice [44], in A3 KO mice it surprisingly reduces plasma TG levels [46]. These paradoxical phenotypes require an explanation.

To explain these phenotypes, the Hobbs laboratory posited that ‘ANGPTL3 tonically suppresses LPL activity in WAT as well as oxidative tissues’ and that ‘circulating ANGPTL3 and ANGPTL8 also inhibit postprandial LPL activity in WAT’ [97]. We disagree.

It is well established that feeding increases WAT LPL activity [91]. If A3 and/or A3/8 inhibited WAT LPL, then feeding would, due to increased plasma A3/8, negatively regulate WAT LPL activity, which is inconsistent with the established conclusion. In other words, if A3 and/or A3/8 inhibited both WAT and oxidative-tissue LPL, it would be difficult to explain the opposite changes of postprandial LPL activities in WAT versus oxidative tissues.

**Figure 3.**
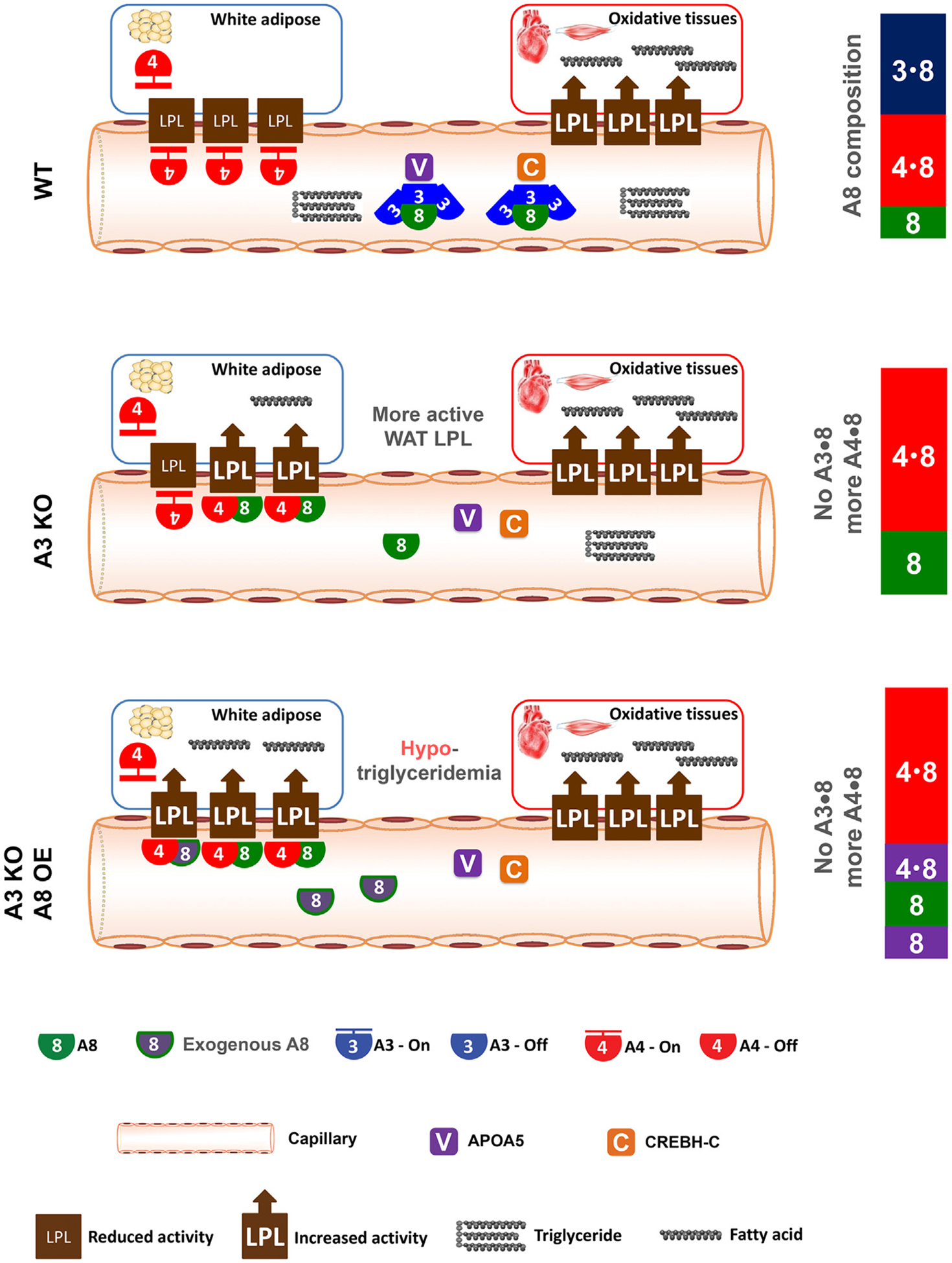
Explanation of the phenotypes of *Angptl3* knockout (KO) mice by the ANGPTL3-4-8 model. During fasting, in wild-type (WT) mice, ANGPTL3/ANGPTL8 (A3/8) and A4/8 are present at comparable concentrations. In A3 KO mice, A3/8 is removed, leading to more availability of A3-free A8, thus increasing the formation of A4/8 and releasing WAT LPL activity. Therefore, WAT LPL activity is increased in A3 KO mice. Further increasing A8 levels by overexpression (OE) leads to more A4/8 and higher WAT LPL activity, and thus reduces triglyceride (TG) levels compared to mice without exogenous A8.

We propose that the increased WAT LPL activity in A3 KO mice stems from elevated WAT A4/8 complexes. In these mice, the absence of A3, and hence the absence of the A3/8 complex, leads to all available A8 existing in an A3-free form. This surplus of free A8 results in increased formation of A4/8 complexes, which exhibit a reduced ability to inhibit LPL compared to A4 alone, hence leading to higher WAT LPL activity (Figure 3, middle panel).

According to the ANGPTL3-4-8 model, we would expect that, in A3 KO mice, further elevation of A8 levels via forced expression would further increase free A8 availability, thereby promoting more A4/8 formation and consequently higher WAT LPL activity. That is, during fasting, LPL activity is high in both WAT and oxidative tissues, resulting in lower TG levels (Figure 3, lower panel). This analysis is consistent with experimental results. That is, in A3 KO mice, forced expression of A8 reduced the serum TG [46]. Therefore, the unexpected phenotypes of A3 KO mice, namely, increased WAT LPL activity and reduced plasma TG with A8 overexpression, are, in fact, consistent with the ANGPTL3-4-8 model (Figure 3). Of note, the aforementioned explanation, although consistent with the experimental results, is still a hypothesis that needs to be validated because, for example, there is still no direct evidence to prove that circulating A8 can affect the amount of the A4/8 complex in WAT.

Some other unexpected phenotypes related to A8 are noteworthy. (i) Unlike WT mice, in A8 KO mice feeding reduces plasma TG levels [55]. (ii) Unlike WT mice, in A8 overexpressing mice fasting increases plasma TG levels [55]. (iii) Unlike A8 whole-body KO mice, adipose-specific A8 KO mice exhibit increased plasma TGs [50]. These seemingly unexpected phenotypes are all consistent with and are explained by the ANGPTL3-4-8 model (Box 1) [8,14].

## Therapeutic potential of ANGPTL3/8 antibodies

APOA5 deficiency, a rare genetic condition, disrupts lipid metabolism and causes abnormal TG levels in the bloodstream. APOA5 is vital for TG breakdown, and its absence leads to hypertriglyceridemia and elevates the risk of cardiovascular diseases. Similarly, CREBH deficiency, another uncommon genetic disorder, affects CREBH functionality and leads to hypertriglyceridemia. Neither APOA5 nor CREBH deficiencies have specific therapies. Typically, medications including fibrates or ω3 fatty acids are prescribed to lower TG levels.

**Figure 4.**
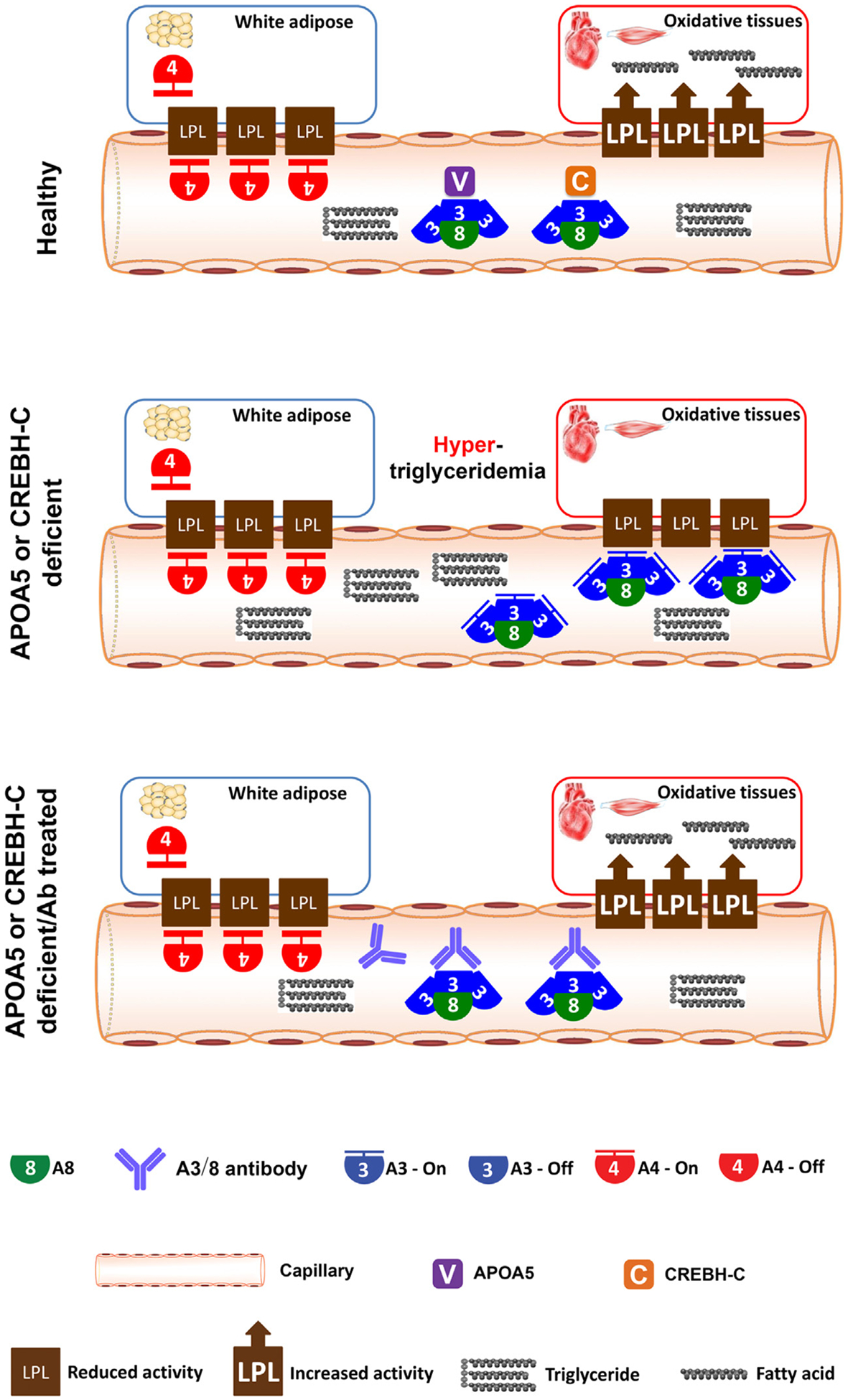
Therapeutic potential of treating APOA5 or CREBH deficiency patients with an antibody (Ab) against ANGPTL3/ANGPTL8 (A3/8). During fasting, APOA5 or CREBH-C deficiency leads to the hyperactive A3/8 complex and hypertriglyceridemia. In these patients, the A3/8 antibody has the potential to correct this condition and normalize plasma triglyceride (TG) levels.

The recent identification of APOA5 and CREBH-C as endogenous inhibitors of A3/8 suggests that the root cause of these two genetic conditions lies in the hyperactive A3/8 complex, especially during fasting states. Consequently, an effective treatment approach for both conditions might be the use of A3/8 antibodies or small-molecule inhibitors (Figure 4).

In the general population, inhibiting A8 has shown promise. Individuals with A8 truncating variants often exhibit low TG levels and high HDL cholesterol levels, leading to a lower incidence of heart diseases, even with minimal statin use [64]. We articulated the evidence that A8 inhibition has the potential to simultaneously lower TG and raise HDL cholesterol levels [98]. As correctly pointed out in an insightful review that compares and contrasts various drug targets for treating hypertriglyceridemia, another advantage of choosing A3/8 is that the relatively low circulating levels of A3/8 (~0.1 nM) make it easier to target using an antibody-based approach compared to some other potential drug targets [9]. Indeed, clinical trials with A3/8 antibody treatments have demonstrated encouraging outcomes, and A3/8 antibodies reduced TGs by 70% and increased HDL cholesterol levels by 26% [61].

In addition to A3/8 antibodies, another potentially promising approach is to selectively knock down the expression of A8 in the liver. This approach will lead to reduced circulating A3/8 but will not affect WAT A8. Recent data showed that liver-specific, but not adipose-specific, deletion of A8 increases intravascular LPL activity and reduces plasma TG levels [50]. We would like to point out that liver-specific knockdown of A8 also has the potential to simultaneously reduce plasma TG and increase plasma HDL-C levels. The rationale that we articulated for using A3/8 antibodies [98] also applies to liver-specific A8 knockdown. A8 is required by A3 for inhibiting LPL, but is not required by A3 for inhibiting EL [99,100]. Therefore, the entire pool of plasma A3 can be classified as A8-associated A3 and A8-free A3, where the former inhibits LPL and the latter inhibits EL [99,100]. Reducing liver A8 leads to reduced circulating A8. Reduced availability of circulating A8 results in fewer A3/8 complexes (reduced LPL inhibition) but more A8-free A3 (enhanced EL inhibition), hence leading to reduced TG but increased HDL-C levels.

Collectively, these results strongly advocate for A8 and A3/8 as promising therapeutic avenues, not only for specific genetic conditions such as APOA5 or CREBH deficiency but also for a broader patient population with hyperlipidemia.

## Concluding remarks

We have reviewed the current understanding of the intricate landscape of LPL regulation by apolipoproteins (including APOA5, APOC3, and APOC2), ANGPTLs (including A3, 4, and 8), and CREBH, in regulating TG partitioning between WAT and oxidative tissues during the feed/fast cycle. To solve the jigsaw puzzle of LPL regulation, which is composed of many moving parts, A8 is a key because it bridges multiple pieces. A8 forms the A3/8 complex, which interacts with APOA5 and CREBH-C; A8 forms the A4/8 complex, which interacts with plasminogen and tPA to generate plasmin that cuts APOC3 and A3/8. We have updated the ANGPTL3-4-8 model, which elucidates the interconnected, interdependent relationships among ANGPTLs, apolipoproteins, CREBH-C, and LPL, and explains the mechanisms by which these proteins are orchestrated, nutritionally and tissue-specifically, to balance TG partitioning. The ANGPTL3-4-8 model was proposed as ‘a general framework for how TG trafficking is regulated’ [14,55]; this framework has remained consistent with evolving experimental data and added details. The model itself and its insightful refinements are clearly the outcome of the combined efforts of numerous laboratories. Future research will hopefully answer some unresolved issues (see Outstanding questions) and integrate additional LPL regulators into the model. We have also explored the therapeutic potential of antibodies or inhibitors targeting the A3/8 complex as a promising avenue for managing hypertriglyceridemia-related genetic conditions such as APOA5 or CREBH deficiencies, as well as for hyperlipidemia in a broader patient population.

The global rise in obesity has cast a negative light on fat, yet it is crucial to recognize that fat metabolism plays a vital role in human health, and TGs themselves are inherently healthy. Problems arise when TG levels become excessively elevated in the circulation, WAT, or muscles, representing hypertriglyceridemia, obesity, or insulin resistance, respectively. Therefore, a fundamental aspect of managing TGs is to maintain balanced partitioning and to rectify aberrations in specific pathological contexts. Developing a clear mechanistic understanding of TG partitioning is imperative for the advancement of drug therapies and their clinical efficacy. In this pursuit, drugs targeting LPL regulators, including A3, A4, A8, A3/8, APOA5, APOC3, and APOC2, are actively under development [9]. These drugs, including monoclonal antibodies, antisense oligonucleotides, small interfering RNAs, and mimetic peptides, aim to address various aspects of TG metabolism. Therefore, it is important to view these drugs not as replacements for one another but as complementary therapeutics, each targeting specific facets of TG metabolism. By doing so, they offer a more comprehensive approach to managing abnormal TG levels and associated metabolic disorders. As our understanding of TG metabolism deepens and with the increasing availability of a range of LPL regulator-based drugs, we anticipate greater precision in controlling TG metabolism in patients, and thus this holistic approach holds promise for improving the management of metabolic syndromes and related conditions.

## Highlights

ANGPTL8 functions as a molecular switch that activates ANGPTL3 but deactivates ANGPTL4 in LPL inhibition.

APOA5 and CREBH-C are fasting-induced, liver-derived endogenous inhibitors of the plasma ANGPTL3/8 complex.

The ANGPTL4/8 complex binds to WAT LPL, recruits plasminogen and tPA, and generates plasmin that cuts LPL inhibitors, including ANGPTL3/8 and APOC3.

The ANGPTL3-4-8 model integrates the roles of ANGPTLs, apolipoproteins, and CREBH-C in tissue-specific LPL regulation during the feed/fast cycle.

ANGPTL3/8 antibodies and inhibitors are promising drugs to treat hypertriglyceridemia.

## Box 1. The ANGPTL3-4-8 model

The model suggests that feeding increases liver A8, forming the A3/8 complex, which, after being secreted, inhibits oxidative-tissue LPL in an endocrine manner, while reducing WAT A4, which inhibits LPL locally. Thus, postprandial LPL activity diminishes in oxidative tissues but surges in WAT, channeling TG to the latter. Fasting does the opposite. Proposed in 2015 [14,55], the model has remained consistent with evolving experimental data with added details. The updated model integrates the roles of ANGPTL3, 4, 8, apolipoproteins, and CREBH in regulating tissue-specific activities of LPL during the feed/fast cycle (Figure 1). The model elucidates intriguing, yet occasionally unexpected, phenotypes observed in mouse models, as exemplified below.

(i) A3 KO mice display exacerbated TG phenotypes in the fed state, despite stable A3 levels [21]. Explanation: A3 is activated by A8, which is feeding-induced [8].

(ii) In A8 KO mice, feeding reduces plasma TG, unlike the response seen in WT controls [55]. Explanation: deletion of A8 leads to an absence of the A3/8 complex, hence constitutively active oxidative‐tissue LPL. Thus, following feeding, A8 KO mice have high LPL activity in both WAT and oxidative tissues, hence lowered plasma TGs [14].

(iii) In A8 hepatic overexpressing mice, fasting increases plasma TG levels, unlike the response seen in WT controls [55]. Explanation: during fasting, exogenous A8 continues to form the A3/8 complex, which constitutively inhibits oxidative-tissue LPL. Thus, during fasting, A8 overexpressing mice have low LPL activity in both WAT and oxidative tissues, and hence have increased plasma TG levels [14].

(iv) A8 whole-body or liver-specific KO mice exhibit lower plasma TGs, but A8 adipose-specific KO mice display elevated plasma TG levels [50]. Explanation: A8 deficiency in WAT leads to the absence of the WAT A4/8 complex, hence more A8-free A4 that inhibits WAT LPL, thus leading to reduced WAT LPL activity and elevated plasma TG levels [8].

(v) Hepatic A8 overexpression dramatically increases plasma TG levels in WT mice, but it reduces plasma TGs in A3 KO mice [46]. Explanation: A3 deficiency leads to an absence of the A3/8 complex, thus increasing the availability of A3-free A8 and enhancing the formation of the A4/8 complex, hence leading to increased WAT LPL activity and reduced plasma TG levels.

## Outstanding questions

Both APOA5 and CREBH-C are fasting-induced liver-derived endogenous inhibitors of the A3/8 complex. What is the relationship between them? Do they compete or cooperate when inhibiting A3/8? Do they bind to the same site on the A3/8 complex?

What is the structural basis by which A8 activates the LPL inhibitory activity of A3, but deactivates that of A4? What is the structural basis by which A3/8 inhibits LPL?

The A4/8 complex interacts with plasminogen and tPA, which are crucial components of fibrinolysis. Does the A4/8 complex play a role in the development of coronary heart diseases that directly result from impaired fibrinolysis?

The first clinical trial of an antibody against A3/8 shows promising results in lowering TG, lowering LDL-C, and increasing HDL-C levels. Can these beneficial effects translate into a reduced incidence of cardiovascular disease? Can this antibody be used to treat APOA5 or CREBH deficiency?
